# Chia Seed Allergy: Emerging Clinical Evidence, Molecular Allergens, and the Unresolved Question of Cross-Reactivity

**DOI:** 10.3390/nu18183027

**Published:** 2026-09-16

**Authors:** Corina Porr, Anca Vidrighin, Alina Camelia Catana, Emi Marinela Preda, Valentin-Cristian Iovin, Gabriela Mariana Iancu, Dana M. Harris, Cosmina Diaconu

**Affiliations:** 1 Allergology Department, Faculty of Medicine, Lucian Blaga University of Sibiu, 550169 Sibiu, Romania; corina.porr@ulbsibiu.ro; 2 Allergology Department, County Clinical Emergency Hospital Sibiu, Bulevardul Corneliu Coposu 2–4, 550245 Sibiu, Romania; 3 Department of Paediatrics, Faculty of Medicine, Lucian Blaga University of Sibiu, 550169 Sibiu, Romania; 4 Hematology Department, Faculty of Medicine, Lucian Blaga University of Sibiu, 550169 Sibiu, Romania; alinacamelia.catana@ulbsibiu.ro; 5 Department of Haematology, County Clinical Emergency Hospital of Sibiu, 550245 Sibiu, Romania; 6 Department of Radiology, Carol Davila University of Medicine and Pharmacy, 050474 Bucharest, Romania; 7 Department of Radiology and Medical Imaging, Foișor Clinical Hospital of Orthopaedics, Traumatology and Osteoarticular TB, 021382 Bucharest, Romania; 8 Doctoral School Department, Victor Babeș University of Medicine and Pharmacy of Timișoara, 300041 Timișoara, Romania; 9 Department III Functional Sciences, Physiology Discipline, Victor Babeș University of Medicine and Pharmacy of Timișoara, 300041 Timișoara, Romania; 10 Centre of Immuno-Physiology and Biotechnologies (CIFBIOTEH), Department of Functional Sciences, Physiology, Victor Babeș University of Medicine and Pharmacy of Timișoara, 300041 Timișoara, Romania; 11 Department of Dermatology, Faculty of Medicine, Lucian Blaga University of Sibiu, 550169 Sibiu, Romania; gabriela.iancu@ulbsibiu.ro; 12 Clinic of Dermatology, County Clinical Emergency Hospital Sibiu, Bulevardul Corneliu Coposu 2–4, 550245 Sibiu, Romania; 13 Department of Internal Medicine, Mayo Clinic, Jacksonville, FL 32224, USA; 14 Department of Nursing and Dental Medicine, Faculty of Medicine, Lucian Blaga University of Sibiu, 550024 Sibiu, Romania; 15 Clinic of Physical Medicine and Rehabilitation, County Clinical Emergency Hospital Sibiu, Bulevardul Corneliu Coposu 2–4, 550245 Sibiu, Romania

**Keywords:** chia seed, *Salvia hispanica*, food allergy, novel food, molecular allergens, sesame allergy, cross-reactivity, IgE-mediated hypersensitivity, anaphylaxis, seed allergy

## Abstract

Chia seed (*Salvia hispanica* L.) is increasingly incorporated into contemporary foods, yet its allergenic potential remains poorly characterized. Published clinical evidence is limited to a small number of case reports, conference reports, and one case series describing immediate IgE-mediated reactions, cutaneous manifestations, and anaphylaxis. Molecular studies have identified candidate allergenic protein families, including 2S albumins, 7S vicilin-like globulins, 11S legumin-like globulins, and oleosins, and have demonstrated IgE recognition and potential cross-reactivity, particularly with sesame. However, molecular cross-recognition does not establish clinically relevant cross-allergy. This structured narrative review synthesizes the available clinical and molecular evidence regarding chia seed allergy, with emphasis on clinical manifestations, candidate allergens, chia–sesame cross-reactivity, diagnosis, and major knowledge gaps. Current evidence supports chia as a potential emerging food allergen but is insufficient to determine prevalence, natural history, reaction thresholds, or individual risk. Standardized diagnostic extracts, validated specific IgE thresholds, component-resolved diagnostics, and chia-specific oral food challenge protocols are currently lacking. Prospective clinical studies and further molecular characterization are required to establish the true clinical significance of chia seed allergy.

## 1. Introduction

Food allergy represents an increasingly important clinical and public health problem, with most epidemiological and mechanistic research traditionally focusing on a relatively restricted group of major allergenic foods. However, changes in dietary habits, globalization of food consumption, increasing interest in plant-based nutrition, and the introduction of novel and alternative protein sources have progressively expanded the spectrum of foods associated with allergic reactions [1,2].

Chia seeds, obtained from *Salvia hispanica* L., a plant belonging to the Lamiaceae family, have become increasingly popular because of their favorable nutritional profile and versatility as a food ingredient. They are rich in polyunsaturated fatty acids, particularly α-linolenic acid, dietary fiber, proteins, minerals, and several bioactive compounds. Chia is now widely incorporated into bread, cereals, yogurt, smoothies, desserts, nutritional bars, plant-based products, protein-enriched foods, and dietary supplements [3,4,5].

In Europe, chia has been evaluated within the regulatory framework for novel foods. Although available safety assessments have generally supported its use under approved conditions, potential allergenicity has repeatedly been identified as an area requiring attention. European Food Safety Authority assessments have specifically acknowledged that allergic reactions to chia may occur and have raised concerns regarding possible cross-reactivity in individuals allergic to foods such as sesame, peanut, and hazelnut [4,5,6,7,8].

Compared with major food allergens, however, chia allergy remains poorly characterized. The first clinical observations consisted largely of individual case reports [9,10]. Subsequent experimental studies demonstrated IgE-binding proteins and cross-reactivity [11], and a more recent clinical series identified several patients with convincing immediate hypersensitivity reactions following chia ingestion [12]. Most recently, detailed proteomic and immunological work has strengthened the biological plausibility of cross-reactivity between chia and sesame proteins [13].

The available evidence therefore suggests an unusual situation: chia is widely used as a nutrient-dense and functionally versatile food ingredient, potentially allergenic protein families have been identified, and severe reactions have been documented, yet epidemiological, diagnostic, and clinical data remain remarkably limited [9,12,13,14].

The aim of this review is to integrate the currently available evidence regarding chia seed allergy, encompassing molecular allergenicity, sensitization mechanisms, cross-reactivity, clinical manifestations, diagnosis, management, food-processing considerations, regulatory aspects, and major knowledge gaps.

## 2. Methods

### 2.1. Literature Search Strategy

A structured narrative literature review was conducted to identify and synthesize the available clinical, molecular, immunological, proteomic, nutritional, and regulatory evidence relevant to chia seed (*Salvia hispanica* L.) allergy. PubMed/MEDLINE was used as the primary bibliographic database. The search was supplemented by targeted searches of Google Scholar and by manual screening of the reference lists of relevant publications to identify additional eligible studies. The final literature search was performed on 17 August 2026.

The search strategy combined terms referring to chia seed with allergy-related keywords, including “chia allergy”, “chia seed allergy”, “Salvia hispanica allergy”, “chia anaphylaxis”, “chia hypersensitivity”, “chia IgE”, “chia allergen”, “chia protein allergenicity”, “chia cross-reactivity”, “chia sesame cross-reactivity”, “chia peanut”, and “chia hazelnut”. Searches were designed to capture both direct clinical evidence of allergic reactions to chia and experimental evidence concerning chia proteins, IgE recognition, and potential cross-reactivity.

### 2.2. Eligibility Criteria and Study Selection

Publications were considered eligible when they provided information directly relevant to the allergenic potential of chia seed. Eligible primary evidence included case reports, case series, clinical observational reports, experimental immunological studies, proteomic investigations, and allergen-characterization studies involving chia seed or chia-derived proteins.

Publications addressing regulatory safety, novel-food allergenicity, food-allergy diagnosis or management, or homologous allergenic proteins from other seeds, nuts, or plant-derived foods were included as secondary or contextual evidence when necessary to interpret the chia-specific findings.

Studies focusing exclusively on the nutritional, metabolic, agricultural, agronomic, or technological properties of chia without direct relevance to allergy or allergenicity were excluded from the core allergological evidence synthesis.

Titles and abstracts were initially screened for relevance, followed by evaluation of the full publication when potentially eligible. Reference lists of included articles were additionally screened to identify relevant publications not retrieved through the initial database searches.

### 2.3. Classification of the Evidence

To improve transparency and avoid overinterpretation of the limited literature, publications were classified according to the type of evidence they provided.

The core chia-specific primary evidence comprised eight unique publications. Six publications provided direct clinical evidence of chia-associated hypersensitivity, including individual case reports, conference case reports/abstracts, and one small case series. Three publications provided molecular, proteomic, or immunological evidence concerning IgE-binding chia proteins, allergen characterization, or potential cross-reactivity. One publication contributed both clinical and molecular evidence and was therefore represented in both evidence categories but counted only once among the eight unique primary publications.

The six clinical publications included reports describing immediate IgE-mediated reactions, cutaneous manifestations, and anaphylaxis. The principal molecular and immunological studies included investigations of IgE-binding proteins, antibody cross-recognition, protein fractionation, proteomic characterization, sequence homology, and experimental chia–sesame cross-reactivity.

Secondary evidence, including reviews, food-allergy guidelines, regulatory assessments, and studies of homologous allergens from sesame, peanut, hazelnut, other seeds, nuts, or legumes, was used only to provide biological, diagnostic, clinical, or regulatory context. Such publications were not considered direct evidence of clinical chia allergy.

### 2.4. Evidence Synthesis

Because the available chia-specific evidence is sparse and heterogeneous and consists predominantly of case reports, conference abstracts, a small case series, and experimental molecular or immunological studies, no formal meta-analysis was performed. The evidence was synthesized narratively, with particular attention to clinical manifestations, molecular allergens, IgE-binding patterns, cross-reactivity, diagnostic approaches, food processing, management, regulatory considerations, and major knowledge gaps.

Throughout the review, direct clinical evidence of chia allergy was distinguished from experimental evidence of sensitization or cross-recognition and from information extrapolated from other food allergens, reviews, guidelines, or regulatory documents. Molecular homology, IgE binding, and in vitro cross-recognition were not considered equivalent to clinically confirmed cross-allergy unless supported by corresponding clinical evidence.

**Figure 1:**
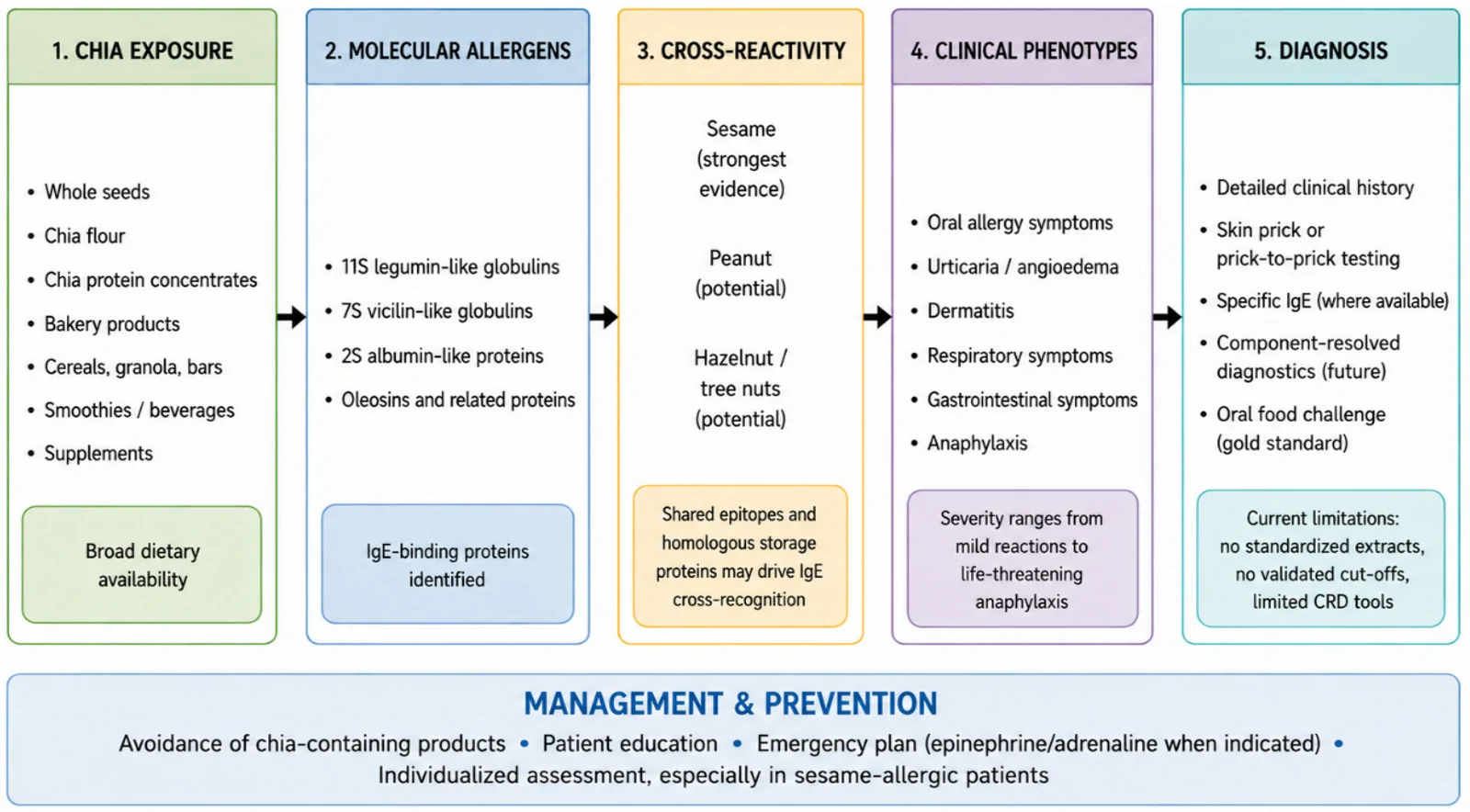
Conceptual overview of chia seed allergy, illustrating the progression from dietary exposure and molecular allergens to cross-reactivity, clinical manifestations, and diagnostic evaluation.

No formal risk-of-bias assessment was performed because of the limited number, heterogeneity, and predominantly descriptive nature of the available primary studies. The study-selection process and classification of the included evidence are summarized in Supplementary Figure S1. During manuscript preparation, OpenAI ChatGPT (GPT-5.6 Sol) was used for language refinement, structural editing, and formatting. OpenAI image-generation tools were also used to assist in producing the conceptual illustration presented in Figure 1 and the study-selection diagram presented in Supplementary Figure S1. All generated outputs were reviewed and edited by the authors, who take full responsibility for the final content.

## 3. Chia as an Emerging Food Source

*Salvia hispanica* L. is increasingly incorporated into contemporary foods, including bakery products, cereals, beverages, plant-based formulations, and dietary supplements. In addition to its lipid and fiber content, chia contains a substantial protein fraction, and modern processing methods allow the production of milled seeds, partially defatted flours, and protein-enriched preparations [3,4,5,13,14].

These developments are relevant from an allergological perspective because concentrated chia-derived ingredients may result in greater exposure to potentially allergenic proteins than whole seeds. However, current evidence does not establish that processed or protein-enriched chia products are clinically more allergenic. Rather, their increasing use broadens opportunities for exposure and may complicate identification of chia as the trigger in multicomponent foods [3,13,14].

Chia should therefore be considered within the broader context of emerging plant-derived food allergens, while recognizing that increased dietary availability does not itself imply an increased prevalence of allergy [1,4,15,16].

## 4. Protein Composition of Chia Seed

Chia seeds contain several protein families with potential allergological relevance. Protein fractionation and proteomic studies have identified seed storage proteins, particularly 11S legumin-like and 7S vicilin-like globulins, together with albumins, oleosins, lipid transfer proteins, and other minor components [13,14].

Among these, 2S albumins, 7S vicilin-like globulins, and 11S legumin-like globulins are of particular interest because homologous proteins are established allergens in several seeds, nuts, and legumes [17,18,19,20]. Recent proteomic analyses of chia have confirmed the presence of these protein families and have demonstrated IgE recognition of globulin fractions and low-molecular-weight proteins tentatively attributed to 2S albumins [13].

Oleosin-related proteins and lipid transfer proteins have also been identified in chia preparations, although their clinical relevance remains uncertain [13,14,21,22]. Overall, these findings support the biological plausibility of chia allergenicity but do not establish that individual chia proteins are clinically validated allergens. Molecular homology and IgE binding should therefore be interpreted as mechanistic evidence rather than proof of clinical reactivity. The principal candidate chia seed protein allergens and their potential allergological relevance are summarized in Table 1.

**Table 1:** Candidate chia seed protein allergens and their potential allergological relevance.

Candidate Protein Family	Main Characteristics/Potential Allergological Relevance	Potential Cross-Reactivity	Current Level of Evidence in Chia
2S albumins	Small seed storage proteins; homologous proteins are recognized as potent and relatively stable allergens in several seeds and nuts	Particularly sesame; potentially other seeds and nuts	Low-molecular-weight IgE-binding chia proteins have been tentatively attributed to 2S albumins; clinical relevance remains unconfirmed
7S vicilin-like globulins	Major seed storage proteins belonging to the cupin superfamily; structural similarities may facilitate IgE cross-recognition	Sesame, peanut, legumes, and other plant foods	Identified in chia by protein and proteomic analyses; molecular homology supports possible cross-recognition, but clinical significance is not established
11S legumin-like globulins	Major seed storage proteins with recognized allergenic homologues in several plant foods	Sesame and other seeds, nuts, and legumes	Identified in chia; IgE-binding and sequence-related similarities have been reported, but predictive clinical relevance is unknown
Oleosins	Hydrophobic proteins associated with seed oil bodies; clinically relevant allergens in some oil-rich seeds and nuts	Potentially other oil-rich seeds and nuts	Oleosin-related proteins have been identified in chia proteomic studies; their clinical role in chia allergy remains unknown
Lipid transfer proteins (LTPs)	Plant-defense proteins associated with allergy to multiple plant-derived foods	Potentially other plant foods containing homologous LTPs	Reported among chia protein fractions, but direct evidence supporting a clinically relevant role in chia allergy is currently insufficient

The proteins listed should be regarded as candidate allergenic protein families rather than fully characterized, clinically validated chia allergens. Molecular homology, IgE binding, and in vitro cross-recognition do not necessarily imply clinically relevant allergy or cross-allergy.

## 5. Molecular Evidence of Chia Allergenicity

Early clinical and laboratory observations demonstrated that chia proteins can bind IgE from affected individuals, providing biological support for IgE-mediated chia allergy [9]. Subsequent experimental work identified antibody-binding chia proteins and suggested immunological cross-recognition with other plant-food allergens [11].

More recently, Calcinai et al. provided the most detailed molecular characterization to date using protein fractionation, high-resolution mass spectrometry, sequence comparison, in silico epitope analysis, and immunoblotting with sera from sesame-allergic patients [13]. Major chia protein fractions included 11S and 7S globulins, albumins, and oleosins, with IgE binding observed in globulin fractions and low-molecular-weight proteins tentatively attributed to 2S albumins [13].

Taken together, these studies support the biological plausibility of chia allergenicity and provide a molecular basis for potential cross-recognition with sesame and other plant foods [9,11,13]. However, the available molecular evidence remains limited in scale and clinical validation. IgE binding, sequence homology, and in vitro cross-reactivity should therefore be interpreted as evidence of sensitization or immunological recognition rather than proof of clinically relevant food allergy. The principal molecular, immunological, and cross-reactivity studies related to chia seed allergenicity are summarized in Table 2.

**Table 2:** Summary of molecular, immunological, and cross-reactivity studies related to chia seed allergenicity.

Study	Methods	Main Molecular/Immunological Findings	Cross-Reactivity	Limitations
García Jiménez et al., 2015 (Spain) [9]	Chia protein extraction; IgE-binding analysis in a clinical case	Several IgE-binding proteins identified, including 11S globulin, lectin and elongation factor; additional proteins remained unidentified	Possible relationship with homologous seed proteins; not systematically investigated	Single patient; limited molecular characterization
Albunni et al., 2019 (Germany) [11]	SDS-PAGE; immunoblotting; sera from 33 hazelnut-allergic and 5 sesame-allergic patients	Several chia protein fractions showed IgG/IgE reactivity and immunological similarities with proteins from other plant foods	Experimental cross-reactivity particularly with sesame and hazelnut; peanut also investigated	In vitro study; small serum groups; cross-reactivity does not prove clinical allergy
Calcinai et al., 2026 (Italy/Germany) [13]	SDS-PAGE; HR-MS; sequence homology; in silico epitope analysis; immunoblotting using sera from two sesame-allergic patients.	Identified 11S and 7S globulins, albumins, and oleosins; IgE binding involved globulin fractions and low-molecular-weight bands tentatively attributed to 2S albumins.	Molecular and immunological evidence supports potential chia–sesame cross-reactivity	Only 2 sesame-allergic sera tested; proof-of-concept in vitro study; incomplete chia sequence data; IgE binding does not establish clinical reactivity

Abbreviations: HR-MS, high-resolution mass spectrometry; IgE, immunoglobulin E; IgG, immunoglobulin G; SDS-PAGE, sodium dodecyl sulfate–polyacrylamide gel electrophoresis.

### Proposed Mechanisms of Chia Seed Allergy

Current evidence suggests that chia allergy may result from primary IgE sensitization to chia proteins or from cross-sensitization to structurally homologous proteins in other plant foods, particularly sesame [9,11,13]. Candidate proteins include 2S albumins, 7S vicilin-like globulins, and 11S legumin-like globulins. Experimental IgE binding and molecular homology support these mechanisms, but their clinical relevance remains incompletely established, and serological cross-recognition should not be equated with clinically confirmed cross-allergy [11,13].

## 6. Cross-Reactivity with Sesame and Other Plant Foods

Among the foods investigated to date, sesame has the strongest molecular and clinical rationale for potential cross-reactivity with chia [11,13,23]. Chia and sesame share homologous seed-storage protein families, including 7S vicilin-like globulins, 11S legumin-like globulins, and low-molecular-weight albumin fractions. Experimental studies have demonstrated IgE recognition of chia proteins by sera from sesame-allergic individuals and have identified molecular similarities that may underlie this cross-recognition [11,13,19,20,23].

Clinical observations provide limited additional support. In the 2023 case series, sesame sensitization was frequent among patients with immediate hypersensitivity to chia, and several had clinically established sesame allergy [12]. A subsequent case report described chia-induced anaphylaxis in a patient with known sesame allergy [24]. However, these findings do not establish the prevalence or clinical predictability of chia reactivity among sesame-allergic individuals.

Potential cross-reactivity with hazelnut, peanut, and other plant foods has also been explored, mainly through experimental antibody-binding studies and molecular homology [11,17,23]. At present, these findings should be regarded as mechanistic evidence rather than proof of clinically relevant cross-allergy.

It is therefore essential to distinguish between IgE cross-binding, cross-sensitization, and clinical cross-allergy. Current evidence is insufficient to support routine chia avoidance in all patients with sesame or other seed and nut allergies. Clinical history and, when appropriate, targeted allergy testing remain necessary to determine the relevance of sensitization at the individual level [11,23,25]. A conceptual overview of the proposed relationships between chia exposure, candidate molecular allergens, cross-reactivity, clinical manifestations, and diagnostic evaluation is presented in Figure 1.

## 7. Clinical Evidence of Chia Seed Allergy

Published clinical evidence of chia seed allergy remains limited but includes immediate IgE-mediated reactions, cutaneous manifestations, and anaphylaxis [9,10,12,24,26,27].

The first well-characterized case was reported by García Jiménez et al. in 2015, describing a systemic reaction after chia ingestion associated with positive skin testing and IgE-binding chia proteins [9]. Tomas-Pérez et al. subsequently reported a patient with eczematous hand dermatitis associated with chia ingestion, positive skin testing, and IgE-binding protein fractions [10].

The largest clinical series to date was reported by Regula et al. in 2023 [12] and included seven adults with immediate hypersensitivity reactions after chia ingestion, including two cases of anaphylaxis. Sesame sensitization was frequent, and several patients had clinically established sesame allergy [12]. Additional reports published in 2024 and 2025 described chia-induced anaphylaxis, including cases in patients with concomitant sesame allergy or sensitization [24,26,27].

Overall, the available clinical literature supports chia as a genuine but insufficiently characterized food allergen. However, the evidence consists predominantly of isolated case reports, conference abstracts, and one small case series. Standardized diagnostic confirmation, including oral food challenge, was not consistently performed. Consequently, current evidence establishes the possibility of clinically significant reactions but does not permit estimation of prevalence, predictive diagnostic thresholds, reaction doses, or natural history [9,10,12,24,26,27]. The published clinical reports are summarized in Table 3.

**Table 3:** Summary of published clinical reports of chia seed allergy.

Study	Country/Type	n; Age/Sex	Clinical Presentation	Diagnostic/Allergy Context	Key Relevance
García Jiménez et al., 2015 [9]	Spain/case report	1; 54/M	Systemic IgE-mediated reaction	Positive chia testing; IgE-binding proteins identified	First combined clinical–molecular report
Tomas-Pérez et al., 2018 [10]	Spain/case report	1; 46/M	Hand eczema/dermatitis	Positive skin prick test; IgE-binding bands at ~60, 30, 15, and 10 kDa	Atypical cutaneous phenotype
Regula et al., 2023 [12]	USA/case-series abstract	7; adults	Immediate hypersensitivity; two cases of anaphylaxis	Six of seven patients sensitized to sesame	Largest clinical series reported to date
Kumar et al., 2024 [24]	USA/conference abstract	1; 41/F	Anaphylaxis	Established sesame allergy; chia testing pending	Clinical signal supporting a possible chia–sesame association
Cortot et al., 2025 [26]	France/conference abstract	1; 16/F	Grade 2 anaphylaxis	Allergic asthma; sensitization profile assessed	One of the few adolescent cases reported
Padin Sobral et al., 2025 [27]	Spain/conference case report	1; 62/M	Anaphylaxis	Positive prick-to-prick testing to chia and sesame; IgE recognition of chia protein bands	Molecular support for potential seed cross-reactivity

## 8. Diagnosis and Differential Diagnosis

Diagnosis of chia seed allergy remains challenging because no standardized chia-specific diagnostic pathway is currently available. Evaluation therefore relies on clinical history, targeted sensitization testing, and, in selected cases, medically supervised oral food challenge [25].

### 8.1. Clinical Assessment

A detailed clinical history is essential and should establish the temporal relationship between chia ingestion and symptoms, the amount consumed, reproducibility, previous tolerance, and potential cofactors [25]. Because chia is often consumed in multicomponent foods, careful assessment of co-ingested ingredients is particularly important. Coexisting sesame or other seed and nut allergies should also be documented, although sensitization to another food does not establish a chia allergy [11,12,23,25].

### 8.2. Skin Testing and Specific IgE

Standardized commercial chia extracts are not routinely available, and published reports have therefore relied mainly on non-standardized extracts or prick-to-prick testing with native chia [9,10,12,27]. Positive skin tests may support sensitization, but their diagnostic performance and clinically meaningful thresholds have not been established.

Similarly, chia-specific IgE is not currently supported by validated decision thresholds for routine clinical interpretation. Experimental studies demonstrate IgE binding to chia proteins, but such findings cannot reliably distinguish between asymptomatic sensitization and clinically relevant allergy [9,10,11,12,13,25].

### 8.3. Molecular Diagnosis

No routinely available component-resolved diagnostic assay currently permits measurement of IgE against individual chia proteins [18,28]. Candidate allergens include 2S albumins, 7S vicilin-like globulins, 11S legumin-like globulins, oleosins, and other proteins identified in molecular studies [13,17,18,19,20,21,22].

Future molecular diagnostics may help distinguish primary chia sensitization from cross-sensitization to sesame or other plant foods. However, individual chia components require further biochemical and clinical validation before they can be incorporated into routine diagnostic algorithms [11,13,23,28].

### 8.4. Oral Food Challenge and Differential Diagnosis

When history and sensitization testing are inconclusive, medically supervised oral food challenge may provide the most direct assessment of clinical reactivity [25]. However, no standardized chia-specific challenge protocol, eliciting-dose distribution, or validated dosing scheme has been established [9,10,12,24,26,27].

Differential diagnosis should consider reactions to co-ingested foods, particularly sesame, peanut, tree nuts, cereals, milk, fruit, and other seeds or pseudocereals, as well as other allergic and nonallergic causes of food-related symptoms [11,23,25,29]. In patients with suspected severe reactions, the indication for oral challenge should be individualized and the procedure performed only in an appropriately equipped clinical setting.

Overall, diagnosis currently depends on the integration of a convincing clinical history with targeted sensitization testing, while avoiding overinterpretation of positive tests or experimental cross-reactivity in the absence of corresponding symptoms [11,13,25,28].

## 9. Influence of Food Processing on Chia Seed Allergenicity

Food processing may alter protein concentration, structure, digestibility, and IgE recognition. This issue is relevant for chia because it is consumed not only as whole seeds but also as milled flour, partially defatted flour, and protein-enriched preparations [13,30].

Calcinai et al. compared several chia preparations and reported substantially higher protein content in a chia protein concentrate than in less processed materials. Immunoblotting with sera from sesame-allergic patients showed stronger IgE-binding signals in the protein concentrate, suggesting that protein enrichment may increase the relative exposure to IgE-reactive proteins [13].

However, these findings are based on in vitro observations and do not demonstrate that processed or protein-enriched chia products are clinically more allergenic. The effects of roasting, baking, grinding, hydration, fermentation, defatting, and gastrointestinal digestion on chia allergenicity remain insufficiently characterized [13,30].

Further studies are needed to determine whether processing-related changes in protein composition or epitope accessibility translate into clinically relevant differences in allergic reactivity.

## 10. Management, Avoidance, and Food Labeling

### 10.1. Clinical Management

No chia-specific management guidelines are currently available. Patients with a convincing diagnosis of chia seed allergy should avoid chia-containing foods and ingredients, with counseling tailored to the severity and certainty of the diagnosis [31].

Because chia may be incorporated into multicomponent foods, plant-based products, bakery items, supplements, and protein-enriched preparations, patients should be advised to review ingredient lists carefully [2,13,31]. Those with a history of systemic reactions should receive an individualized emergency action plan and, when clinically indicated, access to epinephrine auto-injectors in accordance with standard food-allergy and anaphylaxis recommendations [31,32].

Importantly, molecular or serological cross-reactivity alone does not justify indiscriminate avoidance of sesame, peanut, hazelnut, tree nuts, or other seeds. Dietary recommendations should be guided by clinical history, previous tolerance, targeted testing, and, when appropriate, supervised oral food challenge [11,23,25,31].

### 10.2. Food Labeling and Surveillance

Chia is not universally included among regulated priority allergens, and labeling requirements differ between jurisdictions [4,5,6,7,8,15]. Patients may therefore need to rely primarily on general ingredient lists to identify chia-containing products.

Given the limited clinical evidence currently available, there is insufficient basis to determine whether chia should be considered for inclusion among priority allergens. Nevertheless, documented IgE-mediated reactions and anaphylaxis support continued allergovigilance, particularly as chia-derived flours and protein concentrates are incorporated into a broader range of foods [4,5,6,7,8,9,12,13,15,24,26,27].

Future surveillance should document implicated products, reaction severity, coexisting seed allergy, and whether exposure involved whole chia or concentrated chia-derived ingredients.

Chia seed allergy illustrates the broader challenges associated with emerging plant-derived food proteins. As novel and protein-enriched ingredients become more widely incorporated into contemporary diets, assessment of allergenic risk requires integration of molecular characterization, IgE-binding studies, clinical observations, and post-marketing surveillance [1,13,15]. However, experimental evidence of protein homology or IgE recognition should not be interpreted as proof of clinical allergy without corresponding patient-based validation [11,13,25].

## 11. Knowledge Gaps and Future Research Priorities

Major knowledge gaps remain across the epidemiology, diagnosis, molecular characterization, and clinical relevance of chia seed allergy. Current evidence is derived mainly from isolated case reports, conference abstracts, one small case series, and a limited number of molecular and immunological studies [9,10,11,12,13,14,24,26,27].

Population prevalence and natural history are unknown, and pediatric data remain particularly limited [26]. Prospective multicenter cohorts are needed to define demographic characteristics, risk factors, persistence, and the frequency and severity of clinically relevant reactions. More broadly, immune-mediated comorbidities, including autoimmune disorders, have been investigated in related clinical contexts [33,34]; however, no chia-specific evidence currently supports an association between autoimmunity and chia seed allergy.

Further molecular work should characterize the principal chia allergens, define clinically relevant IgE-binding components, and determine the significance of candidate 2S albumins, 7S vicilin-like globulins, 11S legumin-like globulins, and oleosins [13,14,17,18,19,20,21,22]. The clinical relevance of chia–sesame cross-reactivity also requires clarification, with particular emphasis on distinguishing molecular cross-recognition from reproducible clinical cross-allergy [11,12,13,23,24,27].

Diagnostic priorities include development and validation of standardized extracts, chia-specific IgE assays, component-resolved diagnostics, and oral food challenge protocols. Eliciting doses and factors associated with reaction severity, including food matrix, protein concentration, processing, cofactors, asthma, and coexisting seed allergy, also remain to be established [9,12,13,24,25,26,27,28,30].

Finally, structured allergovigilance will be important to determine whether chia allergy remains an uncommon clinical phenomenon or acquires greater relevance as chia-derived ingredients become more widely used in contemporary foods [1,4,5,6,7,8,13,15].

## 12. Conclusions

Chia seed should currently be regarded as a potential emerging food allergen rather than an established major allergen. Available clinical reports demonstrate that *Salvia hispanica* can cause IgE-mediated reactions, including anaphylaxis, but the evidence remains limited to isolated cases, conference reports, and one small case series. Consequently, prevalence, natural history, reaction thresholds, and population-level risk remain undefined.

Molecular studies have identified several candidate allergenic protein families and support a potential immunological relationship between chia and sesame. However, molecular homology, IgE binding, and serological cross-recognition should not be considered equivalent to clinically confirmed cross-allergy.

Future research should prioritize prospective clinical cohorts, standardized diagnostic approaches, molecular characterization of chia allergens, clarification of clinically relevant chia–sesame cross-reactivity, and evaluation of processing and protein concentration. Until such data are available, diagnosis and management should remain individualized and based primarily on clinical history and targeted allergy assessment.

## Data Availability

No new data were created or analyzed in this study. Data sharing is not applicable to this article.

## Supplementary Materials

The following supporting information can be downloaded at: https://www.mdpi.com/article/10.3390/nu18183027/s1, Figure S1: Study selection and evidence classification process for this narrative review.
