# Supplementary material for: Chia Seed Allergy: Emerging Clinical Evidence, Molecular Allergens, and the Unresolved Question of Cross-Reactivity
Source: Nutrients. 2026 Sep 16;18(18):3027. doi: 10.3390/nu18183027 (PMC13610422; doi:10.3390/nu18183027)
Supplement: Supplementary file 1 [file nutrients-18-03027-s001.zip › nutrients-4541061-supplementary.pdf]

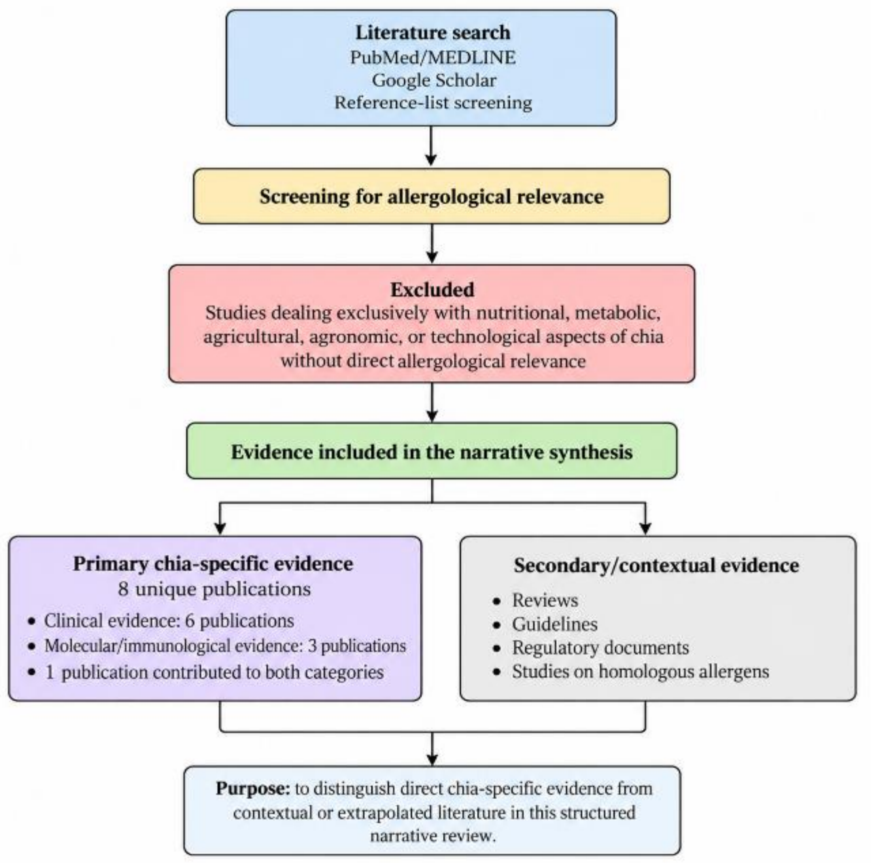

Supplementary Figure S1. Study selection and evidence classification process for this narrative review.
